# Dietary Administration of *Bacillus subtilis* Enhances Growth Performance, Immune Response and Disease Resistance in Cherry Valley Ducks

**DOI:** 10.3389/fmicb.2016.01975

**Published:** 2016-12-08

**Authors:** Mengjiao Guo, Guangen Hao, Baohua Wang, Ning Li, Rong Li, Liangmeng Wei, Tongjie Chai

**Affiliations:** ^1^College of Veterinary Medicine, Sino-German Cooperative Research Centre for Zoonosis of Animal Origin of Shandong Province, Shandong Agricultural UniversityTai'an, China; ^2^Collaborative Innovation Centre for the Origin and Control of Emerging Infectious Diseases of Taishan Medical CollegeTai'an, China

**Keywords:** probiotics, *Bacillus subtilis*, Cherry Valley duck, growth performance, innate immunity, disease resistance

## Abstract

Given the promising results of applying *Bacillus subtilis* (*B*.subtilis) as a probiotic in both humans and animals, the aim of this study was to systematically investigate the effects of *B. subtilis* on growth performance, immune response and disease resistance in Cherry Valley ducks. At 28 d post-hatch (dph), ducks fed a diet with *B. subtilis* weighed significantly more, had higher relative immune organ weights (e.g., bursa of Fabricius, thymus, and spleen), and exhibited greater villus heights, villus height to crypt depth ratios (duodenum and jejunum), and shallower crypt depths in the duodenum than controls fed a normal diet (*p* < 0.05). Moreover, the major pro-inflammatory factors and antiviral proteins, as measured in the thymus and the spleen, were higher at 28 dph in ducks fed probiotics than those of 14 dph. After 28 d of feeding, the ducks were challenged with *Escherichia coli* (*E. coli*) and novel duck reovirus (NDRV), and ducks fed *B. subtilis* achieved survival rates of 43.3 and 100%, respectively, which were significantly greater than the control group's 20 and 83.3%. Altogether, diets with *B. subtilis* can improve Cherry Valley ducks' growth performance, innate immune response, and resistance against *E. coli* and NDRV.

## Introduction

The widespread use of antibiotics as therapeutic agents and growth promoters in animal breeding has increased the antibiotic resistance of bacteria, imbalances of normal microflora, and drug residues in food products on a global scale. In Europe and South Korea, growth-promoting antibiotics have been banned since 2006 and 2012, respectively, and similar bans are expected in other nations as well (Lillehoj and Lee, 2012). One alternative method that has been recommended due to its successful application is the use of probiotics (Reuter, 2001). A previous study has suggested that *Bacillus subtilis* (*B. subtilis*) var. *natto* could be promoted as a biological product intended for humans and animals (Samanya and Yamauchi, 2002).

Also known as probiotics, direct-fed microbials (DFMs) are live microorganisms which have beneficial effects on host health (Reid et al., 2003). Probiotics have been demonstrated to not only modulate symbiotic intestinal bacteria that maintain intestinal homeostasis (Sonnenburg et al., 2006; Martin et al., 2008), but also enhance the barrier functions of intestinal epithelial cells and gut health, as indicated by signs of intestinal morphology such as villus height, crypt depth, and villus height to crypt depth ratio (VH:CD). High villus height and VH: CD ratio correlate directly with increased nutrient absorption and epithelial turnover (Fan et al., 1997). Probiotics can be elicited by antagonizing pathogenic bacteria via the reduction of luminal pH, inhibition of bacterial adherence, and production of antimicrobial molecules (Ng et al., 2009). Studies with pig and poultry fed diets supplemented with probiotic products showed reductions in *Clostridium* and coliform populations in the guts (Choi et al., 2011; Sen et al., 2012), and as expected, probiotics modulated host protective immunity against *Salmonella* infection (Higgins et al., 2011; Levkut et al., 2012).

In poultry, the advantages of probiotics range from improved metabolism, immuno-stimulation, and anti-inflammation to the elimination of pathogens. Healthy carcasses also enhances nutrient absorption and lowers the risk to consumers (Edens, 2003). Innate immunity is a first-line defense against pathogen colonization, and recent findings confirmed that dietary *B. subtilis* can modulate immune response in broiler chickens (Lee et al., 2010). Another recent study showed that *Bacillus*-based DFM significantly induced inflammatory and anti-inflammatory cytokines in the jejunum and ileum of broiler chickens (Rajput et al., 2013).

In poultry, avian colibacillosis induces different syndromes, the most common symptom of which is respiratory disease, as in 3 to 12 weeks old broiler chickens and ducks, usually followed by systemic infection with characteristic fibrinous lesions and fatal septicemia (Schouler et al., 2012). Novel duck reovirus (NDRV) belongs to the genus *orthoreovirus* in the family *Reoviridae*, which includes many viruses, not least the rotavirus, which is a major human pathogen. As observed in China's Fujian, Guangdong, and Zhejiang Provinces, NDRV disease, which caused loss of appetite, unstable gait, diarrhea, and death in various species of ducks (Liu et al., 2011). This disease has been as prevalent as other traditional epidemic diseases, such as avian influenza, duck plague, and ducks Tembusu virus. At the height of their outbreaks, avian colibacillosis and NDRV disease in particular resulted in serious threats to China's duck industry.

According to previous research, we hypothesized that *B. subtilis* can promote growth performance and intestinal morphology in ducks, as well as prevent bacterial and viral infections by stimulating innate immunity. Accordingly, we analyzed the effects of supplementation with DFMs on growth performance, intestinal morphology, innate immunity, and resistance against *Escherichia coli* (*E. coli*) and NDRV in Cherry Valley ducks.

## Materials and methods

### Direct-fed microbials and diet

A *B. subtilis*-based commercial DFM product (Baolai-leelai Biotech Co., Ltd., Tai'an, China) was used in this study. The *B. subtilis* in this product was isolated from cecal contents of healthy chickens. The basal diet was in a non-medicated form (Table 1), which was mixed with DFMs for the DFM diet so that 1 × 10^6^ colony forming units/g (cfu/g) of it comprised *B. subtilis*, per the manufacturer's recommendations. The control diet was formulated by mixing the basal diet with a carrier only.

**Table 1:** **Composition of the experimental basal diet^a^ for Cherry Valley ducks**.

**Ingredients**	**Content**	**Composition**	**Content**
Corn (%)	42	Metabolic energy (MJ/kg)^c^	11.5
Wheat (%)	15	Crude protein (%)	19
Soybean meal (%)	22	Crude fiber (%)	6
Rice bran (%)	8	Crude ash (%)	8
Sunflower meal (%)	7	Sodium chloride (%)	0.5
Cottonseed meal (%)	3	Ca (%)	1.15
NaCl (%)	0.25	P (%)	0.55
Limestone (%)	1	Lysine (%)	0.9
CaHPO_4_ (%)	0.55	H_2_O (%)	14
Lys (%)	0.2		
Premix^b^ (%)	1		
Total	100		

a *The dietary treatments were basal diet supplemented with 0 (control) and 1 × 10^6^ cfu/g B. subtilis*.

b *Supplied per kilogram of diet: 80 mg Fe, 50 mg Mn, 70 mg Zn, 5000 IU vitamin A, 700 IU vitamin D3, 30 IU vitamin E, 0.8 IU vitamin K3, 50 mg niacin, 100 mg pantothenic acid, 5 mg riboflavin, 0.5 mg biotin, 1 mg folic acid*.

c *Calculated values*.

### Culture of pathogens

The bacterial pathogen, *E. coli*, was previously isolated from clinically infected ducks suffering from colibacillosis and stored by the Environmental Microbiology Laboratory at Shandong Agricultural University; its serotype was identified as O1K1 (Li et al., 2016). The bacterial strain was grown in a Luria-Bertani medium at 37°C. The bacterial suspension was prepared to 5 × 10^8^ cfu/mL for a susceptibility study in Cherry Valley ducks.

The NDRV strain used in this experiment was isolated from clinically infected ducks (Yu et al., 2014) and propagated Virus stocks in duck embryo fibroblasts. The viral titer was determined using the method of Reed and Muench (1938), and the virus solution was prepared for the infection experiment at a concentration of 1 × 10^6^ median tissue culture infective doses (TCID_50_)/mL.

### Experimental design

A total of 240 one-day-old Cherry Valley ducks were randomly separated into two treatment groups (DFM or standard control diet). There were three replicates for each treatment with 40 ducks per replicate, all housed in isolators (Table 2). All ducks had free access to feed and water and were handled according to appropriate biosecurity guidelines, and all experimental protocols were approved by the Shandong Agricultural University Animal Care and Use Committee (no. SDAUA-2015-006). The ducks were fed DFM from one day of life. The feeding trial lasted 28 d, during which the ducks' individual body weights were measured at 14 and 28 d post-hatch (dph). On 14 and 28 dph, a total of 15 ducks per treatment (five ducks per replicate) were randomly chosen and euthanized. The spleen, thymus and bursa of Fabricius were weighed and stored at −70°C for RNA extraction. Organ weight was a percentage of body weight. The samples from the middle part of the duodenum, jejunum and ileum were fixed with 4% paraformaldehyde solution to study morphological changes.

**Table 2:** **Experimental design**.

240 ducks	120 control	40	10 Gene
			15 *E. col*i
			15 NDRV
		40	10 Gene
			15 *E. coli*
			15 NDRV
		40	10 Gene
			15 *E. coli*
			15 NDRV
	120 DFM	40	10 Gene
			15 *E. coli*
			15 NDRV
		40	10 Gene
			15 *E. coli*
			15 NDRV
		40	10 Gene
			15 *E. coli*
			15 NDRV

*Gene: 10 ducks per replicate were randomly chosen for the relative expression of genes*.

*E. coli: 15 ducks per replicate were randomly challenged with E. coli*.

*NDRV: 15 ducks per replicate were randomly challenged with NDRV*.

### Small intestinal morphology

The histomorphological measurement of the duodenum, jejunum and ileum was conducted as per the procedure suggested by previous research (Yoon et al., 2012). Briefly, three cross-sections for each intestinal sample were prepared after staining with azure A and eosin using standard paraffin embedding procedures. A total of 10 intact, well-oriented crypt-villus units were selected in triplicate for each intestinal cross-section. All morphological measurements (villus height and crypt depth) were made in 10-μm increments using an image processing and analysis system (Image-Pro Plus version 6, Media Cybergenetics, USA).

### RNA and cDNA preparation

Total RNA was extracted from the spleens and thymuses of ducks using the RNeasy Plus Mini Kit (Qiagen, Carlsbad, CA, USA) according to the manufacturer's instructions. The RNA amount was determined by spectrophotometry (OD 260/280 ratio>1.8). Total RNA (1 μg) was reverse-transcribed with the HiScriptRII One Step RT-PCR kit (Vazyme, Nanjing, China). For controls, the related RT-minus samples were prepared and all products were ultimately stored at −20°C until further use.

### Effect of dietary DFMs on cytokine transcript levels

Quantitative Real-time PCR (qRT-PCR) oligonucleotide primers for cytokines, chemokines, and glyceraldehyde-3-phosphate dehydrogenase (*GAPDH*) as described by Wei et al. (2013) and Li et al. (2014), as presented in Table 3. qRT-PCR was performed using the Applied Biosystems 7500 Fast Real-Time PCR System (Applied Biosystems, CA, USA) with the *TransStart* Tip Green qPCR SuperMix (Transgen Biotech Co., Ltd., Beijing, China) and conducted in a total volume of 20 μL. The qRT-PCR reaction system consisted of 94°C for 30 s, 40 cycles of amplification at 94°C for 5 s, and 60°C for 34 s, followed by a dissociation curve analysis step. The RT-minus controls and negative controls were run on the same plate, and each sample was analyzed in triplicate. The relative expression mRNA was calculated based on the 2^−ΔΔCt^ method and determined using *GAPDH* as an internal reference. The fold changes were logarithmically transformed. The relative expression of immune-related genes were expressed as the probiotic group vs. the control group.

**Table 3:** **Primers used in this study**.

**Gene symbol**	**Sequence(5′–3′)**	**Product size (bp)**	**GenBank no.**
*RIG-1* F	GCTACCGCCGCTACATCGAG	224	EU363349
*RIG-1* R	TGCCAGTCCTGTGTAACCTG		
*MDA5* F	GCTACAGAAGATAGAAGTGTCA	120	KJ451070.1
*MDA5* R	CAGGATCAGATCTGGTTCAG		
*TLR3* F	GAGTTTCACACAGGATGTTTAC	200	JQ910167
*TLR3* R	GTGAGATTTGTTCCTTGCAG		
*TLR7* F	CCTTTCCCAGAGAGCATTCA	150	DQ888645
*TLR7* R	TCAAGAAATATCAAGATAATCACATCA		
*IL-1β* F	TCATCTTCTACCGCCTGGAC	149	DQ393268
*IL-1β* R	GTAGGTGGCGATGTTGACCT		
*IL-8* F	AAGTTCATCCACCCTAAATC	182	DQ393274
*IL-8* R	GCATCAGAATTGAGCTGAGC		
*IL-6* F	TTCGACGAGGAGAAATGCTT	150	AB191038
*IL-6* R	CCTTATCGTCGTTGCCAGAT		
*IL-10* F	CTGACCTCCTACCAGCGAAG	179	NM001310368
*IL-10* R	CTCCATGTAGAACCGCATCA		
*IFN-α* F	TCCTCCAACACCTCTTCGAC	232	EF053034
*IFN-α* R	GGGCTGTAGGTGTGGTTCTG		
*IFN-β* F	AGATGGCTCCCAGCTCTACA	210	KM035791.1
*IFN-β* R	AGTGGTTGAGCTGGTTGAGG		
*IFN-γ* F	GCTGATGGCAATCCTGTTTT	247	AJ012254
*IFN-γ* R	GGATTTTCAAGCCAGTCAGC		
*MX* F	TGCTGTCCTTCATGACTTCG	153	GU202170.1
*MX* R	GCTTTGCTGAGCCGATTAAC		
*OAS* F	TCTTCCTCAGCTGCTTCTCC	187	KJ126991.1
*OAS* R	ACTTCGATGGACTCGCTGTT		
*PKR* F	AATTCCTTGCCTTTTCATTCAA	109	Unpublished
*PKR* R	TTTGTTTTGTGCCATATCTTGG		
*GAPDH* F	ATGTTCGTGATGGGTGTGAA	176	AY436595
*GAPDH* R	CTGTCTTCGTGTGTGGCTGT		

To create the ΔCt_probiotic group_ value, the Ct_probiotic group_ value was normalized to Ct_GAPDH_ _probiotic group,_ or

$$\Delta {\text{Ct}}_{\text{probiotic group}}={\text{Ct}}_{\text{Target mRNA}}-{\text{Ct}}_{\text{GAPDH mRNA}}$$

To create the ΔCt_control group_ value, the Ct_control group_ value was normalized to Ct_GAPDH control group_, or

$$\Delta {\text{Ct}}_{\text{control group}}={\text{Ct}}_{\text{Target mRNA}}-{\text{Ct}}_{\text{GAPDH mRNA}}$$

To create the ΔΔCt value, the ΔCt _probiotic group_ value was normalized to ΔCt_control group_, or

ΔΔCt = (Ct _Target mRNA_ − Ct _GAPDH__mRNA_)_probiotic group_ − (Ct_Target mRNA_ − Ct _GAPDH__mRNA_)_control group._

The relative quantity of target gene mRNA was 2^−ΔΔCt^ (Livak and Schmittgen, 2001).

### Disease resistance experiment

As shown in Table 2, the experiment consisted of two treatments (DFM or standard control diet). 15 ducks were randomly challenged with *E. coli* and NDRV respectively each in triplicate at 28 dph. The bacterial and viral challenge tests were individually conducted in triplicate using respective intraperitoneal and intramuscular injections with 0.5 mL of a stock bacterial suspension or 0.5 mL of a viral suspension containing 2.5 × 10^8^ cfu and 5 × 10^5^ TCID_50_ (Yeha et al., 2008). All experiments were performed in Biosafety Level-2 laboratory. At the end of the experiment, the survival rate of ducks was calculated and all remaining ducks were euthanized via the intravenous administration of sodium pentobarbital (100 mg/kg body weight).

### Statistical analysis

The Student's *t*-test was conducted to examine significant differences among treatments using SPSS 19.0 software (SPSS Inc., Chicago, IL). The significant and highly significant differences were set as *p* < 0.05 and < 0.01, respectively.

## Results

### Growth performance and immune organ index

At 28 dph, ducks fed the *B. subtilis*-supplemented diet were heavier (*p* < 0.05) than the controls. At 14 dph, dietary supplementation with *B. subtilis* had significantly increased the relative weights of the bursa of Fabricius, thymus, and spleen. However, as shown in Table 4, only the relative weight of the bursa of Fabricius increased in response to *B. subtilis* supplementation at 28 dph.

**Table 4:** **Effects of supplementing the diets offered to ducks with ***B. subtilis*** on growth performance and immune organ index**.

	**Control**	**DFM**
**BODY WEIGHT, g/DUCK**		
14 dph	433.0 ± 33.0	448.0 ± 29.7
28 dph	836.7 ± 15.3^a^	1020.0 ± 18.0^b^
**IMMUNE ORGAN INDEX, mg/g**		
Spleen (14 dph)	0.84 ± 0.21^a^	1.26 ± 0.049^b^
Thymus (14 dph)	4.14 ± 0.33^a^	6.31 ± 0.69^b^
Bursa of Fabricius (14 dph)	1.23 ± 0.07^a^	2.74 ± 0.41^b^
Spleen (28 dph)	1.11 ± 0.17	0.98 ± 0.09
Thymus (28 dph)	3.30 ± 0.12	3.40 ± 0.11
Bursa of Fabricius (28 dph)	1.04 ± 0.05^a^	1.35 ± 0.17^b^

*Data were expressed as means ± standard deviations of three replicates (n = 5) per treatment*.

a,b *Values with different superscripts in the same row differ significantly (P < 0.05)*.

*Differences were detected with Student's t test*.

### Small intestinal morphology

Ducks fed the diet supplemented with *B. subtilis* had greater (*p* < 0.05) villus height and VH: CD (duodenum and jejunum) than controls, as well as a shallower crypt depth of the duodenum. As shown in Table 5, the villus height, crypt depth and VH: CD of the ileum of ducks fed the diet supplemented with *B. subtilis* did not significantly differ (*p* > 0.05) from those of the controls.

**Table 5:** **Effects of supplementing the diets offered to ducks with ***B. subtilis*** on small intestinal morphology (28 dph)**.

	**Control**	**DFM**
**DUODENUM**		
Villus height (μm)	902.95 ± 45.49^a^	945.39 ± 26.51^b^
Crypt depth (μm)	213.32 ± 26.09^a^	176.39 ± 16.43^b^
VH/CD	4.29 ± 0.57^a^	5.40 ± 0.61^b^
**JEJUNUM**		
Villus height (μm)	594.65 ± 33.84^a^	689.02 ± 46.32^b^
Crypt depth (μm)	200.22 ± 24.57	180.83 ± 35.90
VH/CD	3.00 ± 0.33^a^	3.92 ± 0.74^b^
**ILEUM**		
Villus height (μm)	426.40 ± 18.00	438.64 ± 27.38
Crypt depth (μm)	129.20 ± 17.89	147.91 ± 30.64
VH/CD	3.36 ± 0.49	3.08 ± 0.66

*Data were expressed as means ± standard deviations of three replicates (n = 5) per treatment*.

a,b *Values with different superscripts in the same row differ significantly (P < 0.05)*.

*Differences were detected with Student's t test*.

### Effect of dietary DFMs on immune-related genes transcript levels of the thymus

**Figure 1:**
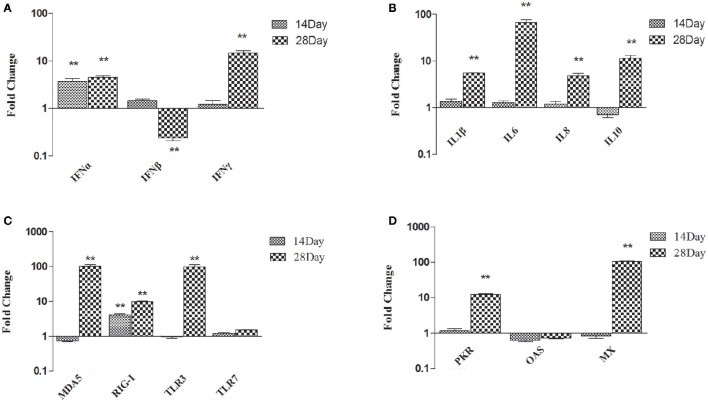
**Expression profiles of immune-related genes in the thymus of ducks. (A)** *IFN*-α, *IFN*-β, and *IFN*-γ **(B)** *IL-1*β, *IL-6, IL-8*, and *IL-10* **(C)** *MDA5, RIG-I, TLR3*, and *TLR7* **(D)** *PKR, OAS*, and *MX*. The Y axis represents the fold change in target gene expression in DFM than that of control group. Data were expressed as means ± standard deviations (*n* = 5). Differences were detected with Student's *t* test and were considered significant as follows: ^**^P < 0.01.

To gain better insight into the role of *B. subtilis*-based DFMs in immune function, the relative expression of immune-related genes in the thymuses of ducks fed the probiotic diet compared to the controls were quantified by qRT-PCR at 14 and 28 dph. The major pro-inflammatory factors and antiviral proteins were up-regulated in ducks fed probiotics and that expressions at 28 dph were higher than those at 14 dph. As shown in Figure 1A, *IFN*-α expression was significantly up-regulated in ducks fed the probiotic diet compared to the controls at 14 dph, by 3.73-fold (*p* < 0.01), whereas the expression of *IFN*-β and *IFN*-γ did not. Moreover, *IFN*-α and *IFN*-γ mRNA expression became up-regulated and peaked at 4.56- and 14.69-fold, respectively, at 28 dph (*p* < 0.01). However, the expression of *IFN*-β down-regulated by 0.24-fold at 28 dph (*p* < 0.01; Figure 1A). At 14 dph, *IL-1*β, *IL-6, IL-8*, and *IL-10* did not obviously change in ducks fed the probiotic diet compared to the controls. *IL-1*β, *IL-6, IL-8*, and *IL-10* were up-regulated significantly at 28 dph compared to 14 dph. Especially *IL-6* was 66.78-fold more expressed at 28 dph than at 14 dph (*p* < 0.01; Figure 1B). At 28 dph, the expression of *MDA5* and *TLR3* exceeded that of other pattern recognition receptors (PRRs). *MDA5* was up-regulated 100-fold in ducks fed the probiotic diet compared to the controls (*p* < 0.01), and *TLR3* mRNA expression up-regulated to reach 97.64-fold (*p* < 0.01). However, the production of *RIG-I* was up-regulated 10-fold at 28 dph (*p* < 0.01; Figure 1C). Antiviral proteins *PKR, OAS*, and *MX* showed no significant difference in ducks fed the probiotic diet compared to the controls at 14 dph. Although *PKR* and *MX* showed elevated expression at 28 dph (12.33-fold and 104.99-fold, respectively; *p* < 0.01), *OAS* continued to show no significant difference (Figure 1D).

### Effect of dietary DFMs on immune-related genes transcript levels of the spleen

**Figure 2:**
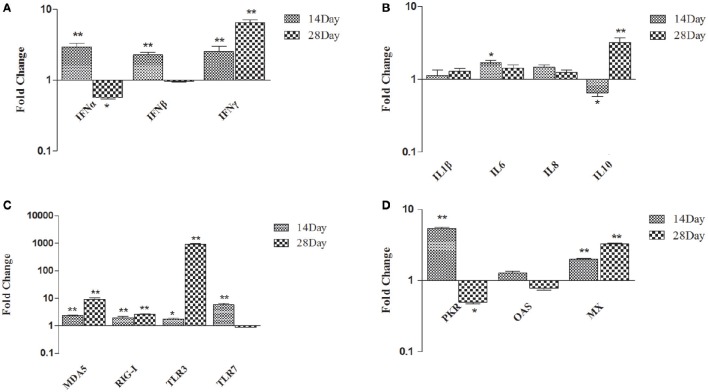
**Expression profiles of immune-related genes in the spleen of ducks. (A)** *IFN*-α, *IFN*-β, and *IFN*-γ **(B)** *IL-1*β, *IL-6, IL-8*, and *IL-10* **(C)** *MDA5, RIG-I, TLR3*, and *TLR7* **(D)** *PKR, OAS*, and *MX*. The Y axis represents the fold change in target gene expression in DFM than that of control group. Data were expressed as means ± standard deviations (*n* = 5). Differences were detected with Student's *t* test and were considered significant as follows: ^*^P < 0.05; ^**^P < 0.01.

In spleen, comparing *B. subtilis*-fed ducks vs. the controls, the expression of type I and II *IFN* became up-regulated at 14 dph (*IFN*-α up-regulated 2.94-fold; *IFN*-β up-regulated 2.28-fold; *IFN*-γ up-regulated 2.54-fold; *p* < 0.01). At 28 dph, *IFN*-γ was up-regulated 6.35-fold (*p* < 0.01) and *IFN*-β showed no significant difference (Figure 2A). At 14 dph, only *IL-6* was up-regulated several fold, whereas *IL-1*β and *IL-8* did not obviously change in ducks fed the probiotic diet compared to the controls. At 28 dph, *IL-10* was up-regulated significantly, while *IL-1*β, *IL-6*, and *IL-8* showed no significant variation (Figure 2B). *MDA5, RIG-I, TLR3*, and *TLR7* had all been up-regulated in ducks fed the probiotic diet compared to the controls at 14 dph, and *MDA5, RIG-I*, and *TLR3* were expressed more frequently at 28 dph (especially *TLR3* up-regulated 920.29-fold, *p* < 0.01), but *TLR7* had no significant variation (Figure 2C). Antiviral proteins *PKR* and *MX* were up-regulated at 14 dph, while the expression of *OAS* demonstrated no significant variation in ducks fed the probiotic diet compared to the controls at either14 or 28 dph (Figure 2D).

### Survival rate

The survival rate of ducks fed *B. subtilis* was significantly higher than that of ducks fed the control diet after the *E. coli* and NDRV challenges. For the *E. coli* challenge test, the survival rate of ducks fed *B. subtilis* was 43.3%, whereas that of the control group was 20% (Table 6). Similarly, for the NDRV challenge test, the survival rate of ducks fed *B. subtilis* was 100%, though that of controls was 83.3% (Table 6).

**Table 6:** **The survival rate of ducks post-challenge with ***E. coli*** and NDRV**.

**Challenge**	**Survival rate(%)**	
	**Control**	**DFM**
*E. coli*	20.0 ± 10.0^a^	43.3 ± 5.8^b^
NDRV	83.3 ± 5.8^a^	100^b^

*Data were expressed as means ± standard deviations of three replicates (n = 5) per treatment*.

a,b *Values with different superscripts in the same row differ significantly (P < 0.05)*.

*Differences were detected with Student's t test*.

## Discussion

The present results indicated that when used in the diet of ducks as a potential growth promoter at hatching, dietary *B. subtilis* can benefit ducks' performance, immune organ index, intestinal morphology, and resistance against *E. coli* and NDRV. Moreover, the expression of primary PRRs in spleen were consistent with results in the thymus. Major pro-inflammatory cytokines and antiviral proteins tended to be up-regulated in ducks fed *B. subtilis*. As concerns the growth promoting effects of *B. subtilis*, the addition of *B. subtilis* spores increased the body weight of the ducks (Xing et al., 2015), and the benefits of *B. subtilis* were also consistent with those detected in piglet and broiler chichens performance studies (Mountzouris et al., 2010; Zhou et al., 2010).

Improved growth performance with probiotics occurs primarily via provided nutrient and enzymatic digestion (Sahu et al., 2008). *Bacillus* sp. can produce certain essential nutrients (e.g., amino acids), vitamins K and B12, and extracellular enzymes (e.g., proteases and lipases), as well as by providing necessary growth factors to promote host growth (Rosovitz et al., 1998; Sanders et al., 2003). Previous research has reported that the relative weights of the liver and bursa of Fabricius were unaffected by diets containing 10^8^ cfu/kg *B. subtilis* in broiler chickens (Zhang et al., 2012). However, another study showed that the relative weight of the spleen increased by adding *B. subtilis* in broiler diets (Awad et al., 2009). The present study indicated that the relative weights of the bursa of Fabricius, thymus, and spleen increased significantly due to dietary supplementation with *B. subtilis* at 14 dph. Such inconsistencies could stem from differences among animal species or among concentrations and species of direct-fed probiotics. Measuring immune organ weight is a common method of evaluating the immune status in chickens (Heckert et al., 2002). Enhanced immune ability might also explain increased body weight gain. In any case, *B. subtilis* exerted some immune-related benefits in ducks.

The crypt is the site of the proliferation and differentiation enterocytes that migrate and boost villus growth (Uni and Perry, 2006). *B. subtilis* promotes shallower crypt depth, which results in longer villi, greater villi surface area and more absorptive epithelial cells. Furthermore, shallower crypt depth promotes rapid epithelial turnover in response to inflammation from pathogenic bacteria (Al-Fataftah and Abdelqader, 2014). Changes in small intestinal morphology and in particular, increased villus height and VH: CD ratio in ducks fed diets supplemented with *B. subtilis* indicate improved gut health and digestive capacity (Caspary, 1992). The results are consistent with the results of broiler chickens fed diets supplemented with *B. subtilis* LS 1-2 (Sen et al., 2012).

The innate immunity system is highly conservative, triggered by the activation of PRRs, and plays an essential role in defending against pathogenic microorganisms (Barbalat et al., 2011). In this study, the expression of *MDA5* and *TLR3* was up-regulated in the thymus and spleen at 28 dph. The activation of PRRs induces the expression of cytokines and antiviral proteins. Cytokines are immune regulatory peptides with relatively small molecular weights that participate in innate and adaptive host immune responses (Lee et al., 2015). Among our results, dietary *B. subtilis* significantly up-regulated pro-inflammatory cytokines *IL-1*β, *IL-6, IL-8*, and *IFN*-γ in the thymus of ducks at 28 dph. *IFN*-γ is a common marker of cellular immunity (Lillehoj and Choi, 1998), whereas *IL-1*β, produced by macrophages, monocytes and dendritic cells, is a major pro-inflammatory cytokine that mediates innate immunity. A recent study moreover revealed that a *Bacillus*-based diet significantly induced inflammatory and anti-inflammatory cytokines in the jejunum and ileum of broiler chickens (Rajput et al., 2013).

Type I *IFN* production is a typical innate defense against viral infection and the expression of antiviral proteins contributes to viral clearance. The expression of *MX* and *OAS* increased in the thymus and spleen of mice infected with West Nile virus (WNV), thereby suggesting that the expression could be involved in protection against WNV (Venter et al., 2005). In this study, *IFN*-α*/*β expression were significantly induced in the thymus and spleen at 14 dph. Accordingly, the expression of *MX, PKR*, and *OAS* increased significantly in the spleen at 14 dph, which suggests that type I *IFN*s can activate the expression of many *IFN*-stimulated genes (*ISGs*), including *MX, PKR*, and *OAS*, which can interfere with virus replication. Although the expression of type I *IFN*s in the thymus was also up-regulated at 14 dph, no significant difference occurred in the *ISGs*, not even a slight down-regulation. In response to that, the underlying mechanisms to this observation should be studied.

To our knowledge, this is the first report of the innate immune response to dietary *B. subtilis* in ducks. *B. subtilis* was recognized by PRRs, which triggers the innate immune response of the host. Moreover, changes in the gut microbiota due to the *B. subtilis* addition may have also led to the different activation of PRRs. In this study, the expression of TLRs and RIG-I-like receptors (RLRs) were up-regulated in the thymus and spleen. Previous research showed that *TLR3* plays a critical role in the expression of pro-inflammatory cytokines, such as *IL-6* and *IL-8* (Le et al., 2007). The activation of *duRIG-I* signaling induces the expression of downstream innate immune genes including *Mx, PKR*, and *IFN*-β (Barber et al., 2013). Since *MDA5* is a strong inducer of antiviral molecules and pro-inflammatory cytokines such as *IL-2, IL-6, IFN*-α, and *IFN*-γ (Wei et al., 2014), the upregulation of cytokines and antiviral proteins expression might have stemmed from the activation of these receptors.

Previous studies have reported that the lipopolysaccharide and bacterial modulins of bacteria induce cytokines (Aldapa-Vega et al., 2016). Bacterial adhesion can induce the release of cytokines. Pathogens are able to not only stimulate host cells to produce cytokines, but also to synthesize proteins or toxins directly or indirectly inhibit the production of cytokines in host cells and cause diseases (Henderson et al., 1996). *B. subtilis* has many beneficial effects on hosts, it activated the immune response of the spleen, thymus and other organs related, and regulated host innate immunity (Lee et al., 2015). However, innate immune responses differed in the thymus and spleen with dietary *B. subtilis* supplementation in our research. Considering the complexity of the mucosal immune system, drawing conclusions about results from immunomodulatory studies tend to be rather difficult. For that reason, increased, decreased, or unchanged gene expression of different cytokines cannot be interpreted strictly as a benefit or detriment (Lähteinen et al., 2014). As such, more detailed information about active crosstalk between supplemented probiotics and host mucosal immune system is clearly necessary to better understand immune-related alterations induced in the ducks gut.

Probiotics enhance the resistance of aquatic animals against pathogens by improving the non-specific immune system as previously described (Nayak, 2010). Dietary administration of lactic acid bacteria significantly increased the survival rate in orange grouper fish or *Epinephelus coioides* challenged with *Streptococcus* sp. and grouper iridovirus (Son et al., 2009), as well as in white shrimp or *Litopenaeus vannamei*, challenged with *Vibrio alginolyticus* (Chiu et al., 2007). The results of the current study indicated that dietary *B. subtilis* immunologically modulated hosts to increase their defense capability against *E. coli* and NDRV infection. The researchers detected that the mRNA expression of *TLR3* and *MDA5* were significantly up-regulated in DFM before the challenge. It is generally known that *TLR3* and *MDA5* can recognize viral dsRNA and activate the *IFN* signal pathway, thereby promoting the production of cytokines (Takeda and Akira, 2005). Since the genome of NDRV is dsRNA, *TLR3*, and *MDA5* might have recognized the NDRV and promoted the antiviral response in the thymus and spleen. Probiotics benefit hosts by producing inhibitory compounds, competing for adhesion sites, stimulating immune function, and improving the microbial balance (Fuller, 1989; Mccracken et al., 1999; Verschuere et al., 2000). As a result, the survival rate of ducks fed *B. subtilis* was higher than that of controls after challenged with *E. coli*. This conclusion takes support from increased body weights and the enhanced expression of major innate immunity genes involved in initiating and regulating immune response against *E. coli* and NDRV in *B. subtilis*-fed ducks compared with those in controls.

In sum, *B. subtilis* improved growth performance, immune response and disease resistance in Cherry Valley ducks. Such awareness of resistance to pathogenic microorganisms caused by probiotics should encourage the development of probiotics, and these results in general inform the relationship of probiotics and host immune response.

## Author contributions

MG and NL designed and conducted the study, performed most of the experiments, and wrote the manuscript. GH and RL performed the calculation with support from TC. LW and BW collected samples. MG and NL performed the biological experiments. TC and LW discussed the results and revise the manuscript.

### Conflict of interest statement

The authors declare that the research was conducted in the absence of any commercial or financial relationships that could be construed as a potential conflict of interest.
